# Functional Porous Ionic Polymers as Efficient Heterogeneous Catalysts for the Chemical Fixation of CO_2_ under Mild Conditions

**DOI:** 10.3390/polym14132658

**Published:** 2022-06-29

**Authors:** Zhifeng Dai, Yang Long, Jianliang Liu, Yuanfei Bao, Liping Zheng, Jiacong Ma, Jiayi Liu, Fei Zhang, Yubing Xiong, Ji-Qing Lu

**Affiliations:** 1 Key Laboratory of the Ministry of Education for Advanced Catalysis Materials, College of Chemistry and Life Science, Zhejiang Normal University, Jinhua 321004, China; 2 Key Laboratory of Surface & Interface Science of Polymer Materials of Zhejiang Province, Department of Chemistry, College of Science, Zhejiang Sci-Tech University, Hangzhou 310018, China; 202130107313@mails.zstu.edu.cn (Y.L.); 202030107252@mails.zstu.edu.cn (J.L.); 202030107214@mails.zstu.edu.cn (Y.B.); 202120104190@mails.zstu.edu.cn (L.Z.); 2020326602050@mails.zstu.edu.cn (J.M.); 2020326602068@mails.zstu.edu.cn (J.L.); 3 Longgang Institute of Zhejiang Sci-Tech University, Wenzhou 325802, China; 4 Institute of Advanced Materials, College of Chemistry and Chemical Engineering, Jiangxi Normal University, Nanchang 330022, China

**Keywords:** porous ionic polymers, CO_2_ elimination, bifunctional heterogeneous catalysts, cycloaddition reaction

## Abstract

The development of efficient and metal-free heterogeneous catalysts for the chemical fixation of CO_2_ into value-added products is still a challenge. Herein, we reported two kinds of polar group (−COOH, −OH)-functionalized porous ionic polymers (PIPs) that were constructed from the corresponding phosphonium salt monomers (v-PBC and v-PBH) using a solvothermal radical polymerization method. The resulting PIPs (POP-PBC and POP-PBH) can be used as efficient bifunctional heterogeneous catalysts in the cycloaddition reaction of CO_2_ with epoxides under relatively low temperature, ambient pressure, and metal-free conditions without any additives. It was found that the catalytic activities of the POP-PBC and POP-PBH were comparable with the homogeneous catalysts of Me-PBC and PBH and were higher than that of the POP-PPh_3_-COOH that was synthesized through a post-modification method, indicating the importance of the high concentration catalytic active sites in the heterogeneous catalysts. Reaction under low CO_2_ concentration conditions showed that the activity of the POP-PBC (with a conversion of 53.8% and a selectivity of 99.0%) was higher than that of the POP-PBH (with a conversion of 32.3% and a selectivity of 99.0%), verifying the promoting effect of the polar group (−COOH group) in the porous framework. The POP-PBC can also be recycled at least five times without a significant loss of catalytic activity, indicating the high stability and robustness of the PIPs-based heterogeneous catalysts.

## 1. Introduction

The transformation of CO_2_ into industry products seems to be an attractive way for the elimination of CO_2_ and relieving the effect of global warming [1,2,3]. Nowadays, CO_2_ could be readily transformed into fruitful products, including CO [4], formic acid [5], CH_4_ [6,7], methanol [8,9], cyclic carbonates [10,11,12,13], and so on [14]. Due to the relatively green process and high atom-economy, as well as the wide application of the cyclic carbonates in the industry process, the cycloaddition of CO_2_ with epoxides to form cyclic carbonates have attracted much attention recently, although the market size for cyclic carbonates is still limited (300–400 kta) compared with the emission of 40 Gta of CO_2_ [11,15,16,17,18,19]. Nowadays, various catalysts, including homogeneous [11,17,20] and heterogeneous [21,22,23,24,25] catalysts have been developed for the cycloaddition reaction to form cyclic carbonates. However, most of these catalysts are homogeneous and severe conditions, such as high temperature and high pressure or extra additives served as co-catalysts are always required [22,26]. Obviously, the utilization of homogeneous catalysts or additives will complicate the catalysts’ separation and product purification; the separation processes of the catalysts from the reaction mixture are energy-intensive [27,28,29]. In this case, the development of novel heterogeneous catalysts has drawn the attention of many researchers. Otherwise, little research has focused on the transformation of low concentration CO_2_ [30]. Therefore, the development of highly efficient heterogeneous catalysts which can promote the coupling reaction under relatively mild and co-catalyst-free conditions are essential.

Recently, various novel porous materials, including metal–organic frameworks (MOFs), covalent organic frameworks (COFs), and porous organic polymers (POPs) have been developed and show fruitful applications, including catalysis, sensor, gas capture and separation, due to their unique porosity structure and the tunability of the chemical structure [31,32,33,34,35]. Among them, porous organic polymers (POPs), which always have special hierarchical porosity, high stability, and a relatively high surface area, are good candidates for heterogeneous catalysis [36,37,38,39,40,41]. Porous ionic polymers (PIPs), as one of the POPs which are constructed from the organic ionic compound of monomers, are potential players for the transformation of CO_2_ into cyclic carbonates, due to their intrinsic ionic structure and CO_2_-philic properties [42,43,44,45]. Through a copolymerization method, Han and coworkers [46] prepared an ionic liquid polymer catalyst from imidazolium salt and divinylbenzene, which could promote the cycloaddition reaction of CO_2_ with epoxides under co-catalyst free conditions, although a relatively higher reaction temperature and higher CO_2_ pressure were needed. Wang et al. [47] decorated the imidazolium salt with a hydroxyl electrophilic center, and the catalytic performance of the resulting catalyst was remarkably improved (the reaction can be readily promoted at atmospheric CO_2_ at 70 °C). The excellent performance of these catalysts was attributed to the functional polarity structure of the polymers, as well as the porous structure [43,48,49,50,51]. Different from those copolymerization strategies, Sun et al. synthesized a series of PIPs from the corresponding vinyl-functionalized phosphonium salt monomers which can be used as robust and efficient heterogeneous catalysts for the transformation of atmospheric CO_2_ into cyclic carbonates under metal-free conditions at 298 K without any additives [52].

In this work, we reported two kinds of polar group (−COOH, −OH)-functionalized PIPs that were constructed from the corresponding vinyl-functional phosphonium salts through a solvothermal radical polymerization method (noted as POP-PBC and POP-PBH, respectively). The resulted PIPs can be used as heterogeneous catalysts for the cycloaddition reaction of CO_2_ with epoxides under ambient conditions. The catalytic activities of the heterogeneous catalysts are comparable with those of the homogeneous monomer catalysts (MePBC and PBH). The catalysts outperform those that are synthesized from the post-modification method, demonstrating the importance of high concentration catalytic active sites in the heterogeneous catalysts. It was also found that the POP-PBC was more active than the POP-PBH (with a conversion of 53.8% versus 32.3%), indicating the more promotive role of the −COOH group than the −OH group. The POP-PBC catalyst could be fully reused at least five times without a significant loss of catalytic activity, indicating the high stability and robustness of the PIPs-based heterogeneous catalysts.

## 2. Materials and Methods

### 2.1. Materials

THF was dried using LiAlH_4_. Other reagents and solvents, including 4-bromostyrene, bromoacetic acid, 2-bromoethanol, azobisisobutyronitrile (AIBN), epichlorohydrin, propylene oxide, 1,2-epoxybutane, 1,2-epoxyhexane, and styrene oxide were all commercially available and used without further purification.

### 2.2. Catalyst Preparation

Synthesis of the POP-PBC. The POP-PBC was synthesized from the v-PBC monomers through a solvothermal polymerization method. Typically, 1.0 g of v-PBC was dissolved in 10 mL of DMF, followed by the addition of 50 mg of AIBN. After maintaining in an autoclave at 373 K for 24 h, the product with a light-yellow color was finally obtained after washing with CH_2_Cl_2_ and drying under vacuum for 12 h to give the POP-PBC (0.96 g, 96% yield).

Synthesis of the porous ionic polymer POP-PBH. The POP-PBH was synthesized from the v-PBH monomers through a solvothermal polymerization method. In a typical run, 1.0 g of v-PBH was dissolved in 10 mL of DMF, followed by the addition of 50 mg of AIBN. After maintaining in an autoclave at 100 °C for 24 h, the product POP-PBH was finally obtained as a white powder after washing with CH_2_Cl_2_ and drying under vacuum for 12 h (0.98 g, 98% yield).

### 2.3. Catalytic Activity Test

Typically, in a Schlenk tube with 10 mmol of epoxides, the catalysts were added and the CO_2_ was purged with a balloon and reacted at 333 K for 48 h. The conversion and selectivity were determined by ^1^H NMR spectroscopy. For catalytic evaluation under a low CO_2_ concentration, 15% CO_2_ mixed with 85% N_2_ in volume was used. For the recycling tests, the catalyst was filtered and washed with CH_2_Cl_2_ three times and dried in air. The catalyst was then used for the next run directly.

### 2.4. Characterizations

Nitrogen sorption isotherms of the various materials were collected at Micromeritics ASAP 2020M, and the samples were dried under vacuum at 373 K for 12 h. The surface areas were calculated from the sorption isotherms using the Brunauer–Emmett–Teller (BET) method. CO_2_ sorption isotherms were performed on the Micromeritics ASAP2010 under 1 atm CO_2_ at 298 K and 273 K. The samples were also treated under vacuum at 373 K for 12 h. An X-ray photoelectron spectroscopy (XPS) was performed on a Thermo ESCALAB 250 with Al Kα irradiation at θ = 90° for X-ray sources; the binding energies were calibrated using the C1s peak at 284.9 eV. The Fourier transform infrared (FTIR) spectra were measured on a Nicolet iS10 (Thermo Fisher, Waltham, MA, USA) IR spectrometer in the range of 400–4000 cm^−1^. A thermal gravimetric analysis (TGA) was performed on a SDT Q600 V8.2 Build100 thermogravimetric analyzer under N_2_ flow. An elemental analysis was carried out in the vario MACRO cube organic element analyzer (Elementar, Frankfurt, Germany). The scanning electron microscopy (SEM) images of the samples were recorded on Hitachi SU 1510 apparatus. Transmission electron microscopy (TEM) experiments were performed on a JEM-2100F field emission electron microscope (JEOL, Tokyo, Japan) with an acceleration voltage of 110 kV. ^1^H NMR spectra were recorded on a Bruker Avance-400 (400 MHz) spectrometer. Chemical shifts were expressed in ppm downfield from TMS at δ = 0 ppm. ^13^C and ^31^P magic-angle spinning (MAS) NMR spectra were carried out on a Varian infinity plus 400 spectrometer with a magic-angle spin probe in a 4-mm ZrO_2_ rotor.

## 3. Results

**Scheme 1:**
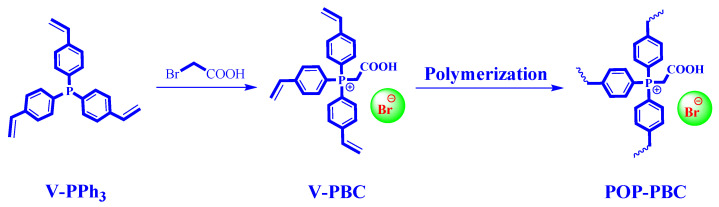
Illustration of the synthesis process of PIPs from the vinyl-functionalized monomer.

Porous ionic polymers POP-PBC and POP-PBH are prepared using a solvothermal radical polymerization method from the corresponding vinyl-functionalized phosphonium salts monomers, and the polymers were finally obtained in nearly quantitative yields after the removal of the solvents and drying under vacuum (as shown in Scheme 1 and the Experimental Section). The vinyl functional phosphonium salts monomers (v-PBC and v-PBH) were synthesized accordingly through the facile phosphorylation reactions of bromoacetic acid and 2-bromoethanol, respectively, with the tri(4-vinylphenyl) phosphine. For comparison, the heterogeneous catalyst POP-PPh_3_-BC that was constructed using a post-modification method and a series of homogeneous catalysts, including (carboxymethyl)tri-p-tolylphosphonium bromide (Me-PBC) and (2-hydroxyethyl) triphenylphosphonium bromide (PBH) was synthesized. It is worth mentioning that the attempt to synthesize (carboxymethyl)triphenylphosphonium bromide (PBC) failed, which is mainly due to many side effects of the phosphorylation reaction of triphenylphosphine with bromoacetic acid (Scheme S1), according to the literature [53]. However, when the tri(4-methylphenyl) phosphine was introduced, the Me-PBC could be obtained with high purity (Figure S3). Therefore, the Me-PBC was used instead of the PBC. The synthesis processes and characterization details of Me-PBC and PBH are listed in the Supplementary Materials.

**Figure 1:**
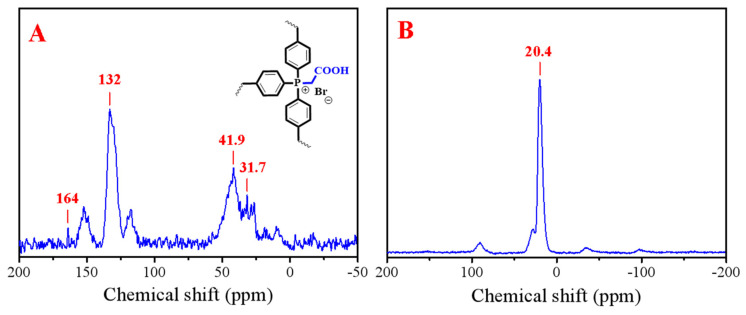
(**A**) ^13^C and (**B**) ^31^P solid state NMR spectrum of the POP-PBC.

The chemical structure and composition of the polymers were evaluated by the solid-state NMR spectroscopy and FT-IR spectroscopy, and the POP-PBC was used as a representative material for further discussion. Figure 1A shows the ^13^C solid state NMR spectra of the POP-PBC. The main peaks are at around 31.7, 41.9, 119, 132, 150, and 164 ppm, which are almost the same with that of the v-PBC monomer (as show in Figure S1), indicating that the structure of the monomer was well maintained after the polymerization reaction. The peaks at around 110 ppm that were related to the vinyl groups disappeared, and a series of new peaks appeared at about 41.9 ppm, which is assigned to the alkyl groups from the radical polymerization, confirming the quantitative polymerization of the vinyl group. Figure 1B shows that the ^31^P NMR spectra of the POP-PBC and the sharp peak at about 20.4 ppm finely coincided with the phosphonium salt monomer, which further confirms the maintenance of the phosphonium salts monomer in the polymer after the polymerization reaction.

These results were further confirmed by the FTIR spectroscopy of the POP-PBC and v-PBC. As shown in Figure S5, the peak at 1500 cm^−1^ belonging to the vinyl group of v-PBC disappeared in the spectrum of POP-PBC, implying the successful polymerization of the vinyl group under the solvothermal condition. The peak at 1700 cm^−1^ that was attributed to the free carboxyl group in the spectrum of POP-PBC and v-PBC confirms the sound maintenance of the polar group in the phosphonium salt after radical polymerization. Similar results are also observed in the POP-PBH as show in Figure S6.

**Figure 2:**
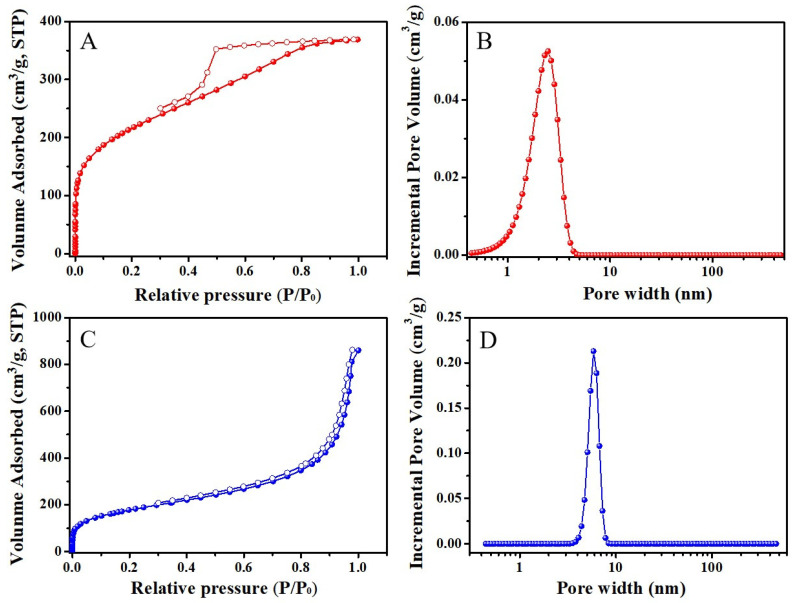
(**A**,**C**) N_2_ sorption isotherm and (**B**,**D**) pore size distribution of POP-PBC and POP-PBH.

The pore structures of the PIPs were determined by the N_2_ sorption tests. As shown in Figure 2A, the N_2_ sorption isotherm of the POP-PBC collected at 77 K shows a typical type I and type IV curve. The rapid increase in the isotherm at a relative pressure (P/P_0_) of below 0.1 is due to the existence of micropores, and the obvious hysteresis loop at a relative pressure (P/P_0_) of higher than 0.40 is due to the contribution of mesopores, indicating the hierarchical structure of the PIPs materials. The nonlocal density functional theory calculation shows that the pore size distribution of the POP-PBC was mainly at 2.5 nm (Figure 2B). The BET surface area of the POP-PBC is calculated to be 772 m^2^/g, with a total pore volume of 0.57 cm^3^/g. For the POP-PBH, the BET surface area is calculated to be 643 m^2^/g, with a total pore volume of 1.3 cm^3^/g (Figure 2C). The pore size was mainly distributed at 5.8 nm (Figure 2D). It has been demonstrated that the high surface area and porosity structure of the heterogeneous catalysts are beneficial for the reagents diffusion and thus could promote the catalytic activities. The N_2_ sorption isotherm of POP-PPh_3_-BC and the corresponding textural parameters are presented in Figure S7 and Table S1.

**Figure 3:**
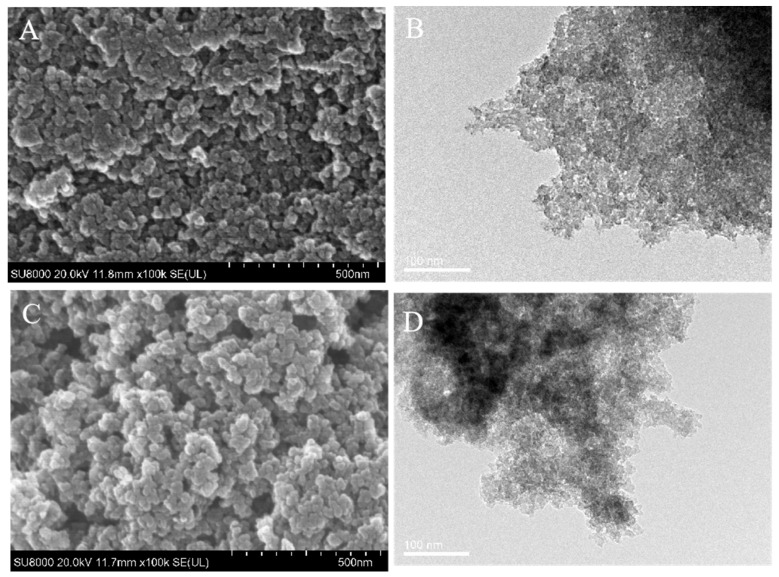
(**A**,**C**) SEM image, and (**B**,**D**) TEM image of (**A**,**B**) POP-PBC and (**C**,**D**) POP-PBH.

Figure 3 shows the SEM and TEM images of the POP-PBC and POP-PBH. As shown in Figure 3A,C, both POP-PBC and POP-PBH are composed of spherical particles and show amorphous morphologies. These results are also confirmed by the TEM images (Figure 3B–D). A thermogravimetric analysis (TGA) was used to test the stability of the PIPs. As show in Figure S8, both the POP-PBC and POP-PBH are stable up to 473 K, which suggests they are stable enough for the coupling of CO_2_ under nearly ambient conditions.

**Figure 4:**
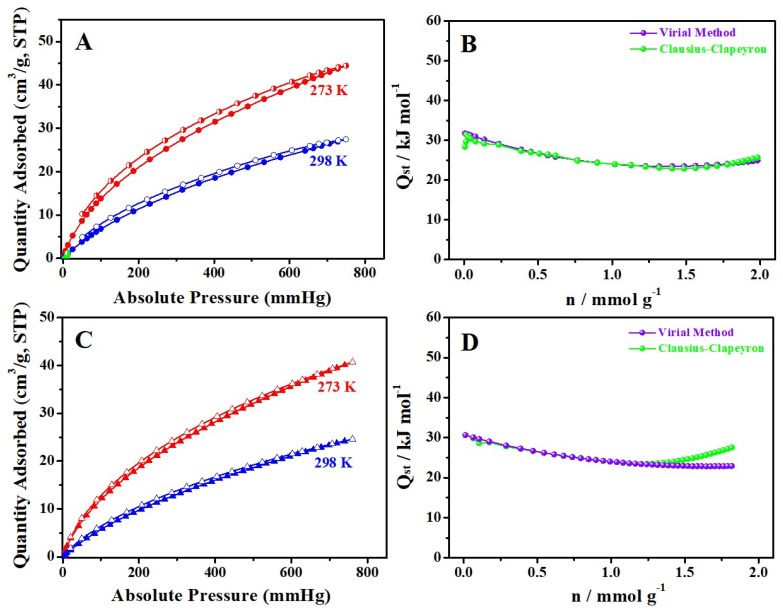
CO_2_ sorption isotherms (**A**,**C**) and the corresponding Qst (**B**,**D**) of POP-PBC (**A**,**B**) and POP-PBH (**C**,**D**) measured at different temperatures.

The CO_2_ affinity and capture property of the PIPs were characterized by CO_2_ adsorption tests at different temperatures of 273 and 298 K under 1 bar CO_2_. As shown in Figure 4A, the CO_2_ adsorption capacity of the POP-PBC at 273 K is 1.98 mmol/g (87 mg/g), which slightly decreases to 1.23 mmol/g (54 mg/g) at 298 K. For the POP-PBH, the CO_2_ adsorption quantity is a little lower than that of the POP-PBC, giving values of 1.82 and 1.10 mmol/g at 273 and 298 K, respectively (Figure 4C). These values are comparable to other porous materials that were reported previously (Table S2) [26,54,55]. The isosteric heat of the CO_2_ adsorption (Qst) of POP-PBC that is calculated from the isotherms using the Virial method is about 31.7 kJ/mol at zero coverage (Figure 4B), which is also higher than that of the POP-PBH (30.6 kJ/mol, Figure 4D) and is comparable to those of other porous organic polymers that are used for CO_2_ capture. Obviously, the relatively high adsorption capacity and moderate values of Qst is helpful to the CO_2_ activation and transformation in the reaction process. The CO_2_ sorption isotherm of POP-PPh_3_-COOH is presented in Figure S9; the Qst was calculated to be 29.5 kJ/mol.

These catalysts were tested for the cycloaddition reaction of CO_2_ with epoxide under solvent-free conditions at a relatively low temperature (313–333 K) and 1 atm pressure of CO_2_ by using high boiling points of epichlorohydrin as typical substrates instead of the propylene oxide. As shown in Table 1, with a catalyst loading of 0.5 mol.%, the catalytic activity of POP-PBC is relatively low at 313 K (with a yield of 35.1%, Table 1, entry 1) after 48 h reaction, and it can be optimized to 75.8% (Table 1, entry 2) when the temperature increases to 333 K. For the POP-PBH catalyst, the yield is about 76.8% at a catalyst loading of 0.5 mol% (Table 1, entry 3). The almost complete transformation of the substrate could be obtained when the loading amount of POP-PBC and POP-PBH were increased to 1.0 mol% (yields of 96.2% and 97.2%, respectively, Table 1, entry 4 and 5). These results are comparable with or higher than those of the literature-reported ionic liquids polymer-based heterogeneous catalysts [26,56,57] and MOF based bifunctional heterogeneous catalysts [46,58,59] (Table S3). It should also be noted that the catalytic activity of the POP-PBC is comparable to that of Me-PBC (with a yield of 86.2%, Table 1, entry 6). Furthermore, the heterogeneous catalyst POP-PPh_3_-BC that was synthesized from the post-modification of POP-PPh_3_ with bromoacetic acid was also illustrated in this transformation as a control experiment. Under the same conditions, the yield on the catalyst POP-PPh_3_-BC (45.7%, Table 1, entry 7) is lower than that on the POP-PBC. We hypothesize that it may be attributed to the POP-PBC that was synthesized from the polymerization of v-PBC, which possesses a relatively higher concentration of the catalytic active sites than that of POP-PPh_3_-BC.

**Figure 5:**
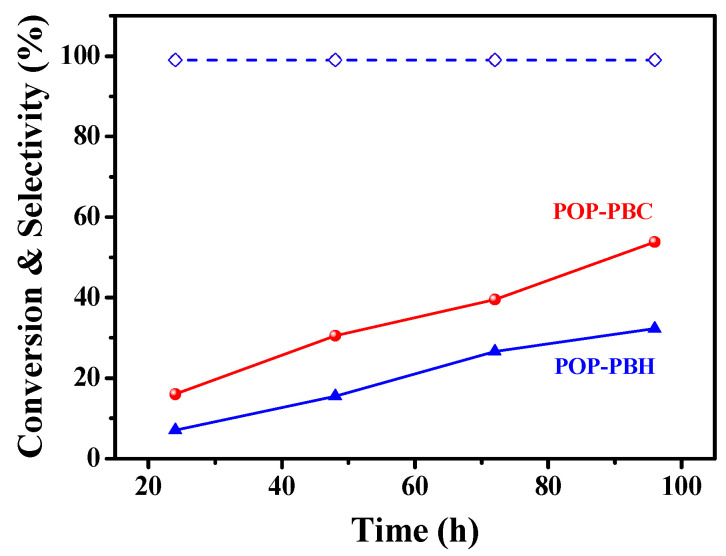
The catalytic performance of the heterogeneous catalysts POP-PBC and POP-PBH in the cycloaddition of CO_2_ with epoxide under low CO_2_ concentration (15% CO_2_ and 85% N_2_, *v*/*v*) condition. Reaction conditions: epichlorohydrin (925 mg, 10 mmol), 1.0 mol.% of catalysts were used under low CO_2_ concentration (15% CO_2_ and 85% N_2_, *v*/*v*) condition at 333 K for 96 h.

We further explored their catalytic activity under low concentrations of CO_2_, as it is known that the concentration of CO_2_ in the industrial exhaust gases is about 7–15 vol.%. Until now, the development of novel heterogeneous catalysts for low concentration CO_2_ elimination without the soluble additives is still rare and remains challenging. Therefore, we further tested the conversion of these functional porous organic polymers as heterogeneous catalysts at low CO_2_ concentrations with epichlorohydrin as the substrate. As shown in Figure 5, after a reaction time of 96 h the conversion of epichlorohydrin over the POP-PBC is 53.8% with a selectivity of 99.0%, which is higher than that of POP-PBH (with a conversion of 32.3% and 48.9% and a selectivity of 99.0%). Considering their similar catalytic sites and the same catalyst loadings, the excellent activity of the POP-PBC could be attributed to the higher uptake capacity and affinity of POP-PBC with CO_2_.

**Figure 6:**
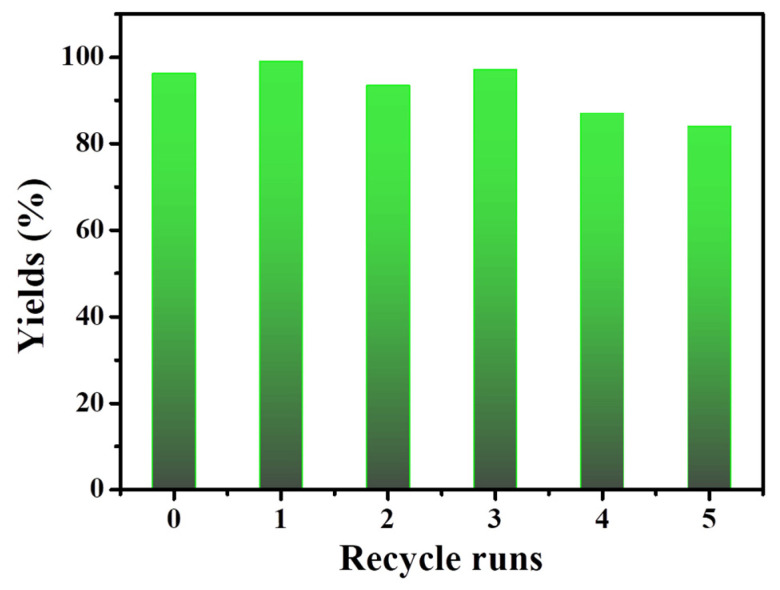
Recycle test of the catalyst POP-PBC in the cycloaddition reaction of the CO_2_ with epichlorohydrin.

A recycle test was carried out to test the reusability and stability of the catalysts. As shown in Figure 6, the POP-PBC could readily recycle five times without significant loss of the catalytic activity. The FT-IR spectrum of the POP-PBC catalyst after recycling five times show that its structure is almost consistent with the fresh POP-PBC (Figure S11), suggesting the robustness of the PIPs-based heterogeneous catalysts.

We also explored the catalytic activity of the POP-PBC and POP-PBH with various substrates. As show in Figure S12, propylene oxide, 1,2-epoxybutane, 1,2-epoxyhexane, and styrene oxide were tested. The catalytic activity decreased dramatically with the increasing molecular size of the substrates, suggesting that the POP-PBC and POP-PBH have an excellent molecular size for use as selective heterogeneous catalysts. Based on the current results, as well as the previous reports, the proposed catalytic mechanism of POP-PBC and POP-PBH in the cycloaddition reaction of CO_2_ with epoxides is illustrated in Figure S13. Firstly, through the hydrogen bonding between the polar group in POP-PBC or POP-PBH and the O atom of epoxides, the substrate is activated and an intermediate is formed through a nucleophilic attack of Br^−^ anion to the epoxide to open the epoxy ring from the carbon atom with less steric resistance. Then, CO_2_ is inserted into the oxygen anion of the open epoxy ring to form the halocarbonate. Finally, the cyclic carbonate is obtained from the corresponding ring-closing step and the heterogeneous catalyst is ready for the next catalytic cycle.

## 4. Discussion

We have synthesized two kinds of polar group-functionalized porous ionic polymers (PIPs) using a solvothermal radical polymerization method from the corresponding phosphonium salt monomers (v-PBC and v-PBH). The resulting PIPs (POP-PBC and POP-PBH) can be used as efficient heterogeneous catalysts in the cycloaddition reaction of CO_2_ with epoxides under relatively low temperature and ambient pressure. The catalytic activities of the POP-PBC are comparable with those of the homogeneous catalysts (MePBC and PBH), indicating the importance of polar group and high catalytic active sites. Moreover, the POP-PBC catalyst can be fully reused at least five times without obvious loss of the catalytic activity, showing the high stability of the heterogeneous catalyst. However, due to the complex synthesis process of those catalysts, as well as the expensive precursors, the practical applicability of these materials is still a problem and the development of a facile post-modification method for the preparation of these heterogeneous catalysts is currently underway in our lab.

## Figures and Tables

**Table 1:** Catalytic performance of different catalysts in the cycloaddition of CO_2_ with epichlorohydrin to form cyclic carbonate ^1^.

Entry	Catalysts	Molar Ratio (mol.%)	Temp. (°C)	Yield (%) ^2^	TON
1	POP-PBC (24.0 mg)	0.5	40	35.1	70.2
2	POP-PBC (24.0 mg)	0.5	60	75.8	151.6
3	POP-PBH (23.3 mg)	0.5	60	76.8	153.6
4	POP-PBC (48.0 mg)	1.0	60	96.2	96.2
5	POP-PBH (46.6 mg)	1.0	60	97.5	97.5
6	MePBC (18.2 mg)	0.5	60	86.2	172.4
7	PBH (19.4 mg)	0.5	60	80.6	161.2
8	POP-PPh_3_-BC (35.7 mg)	0.5	60	45.7	91.4

^1^ Conditions: epichlorohydrin (925 mg, 10 mmol), catalyst (molar ratio was based on the ionic liquid molecular), 1 atm of CO_2_, 48 h. ^2^ Determined by liquid NMR.

## Data Availability

Not applicable.

## Supplementary Materials

The following supporting information can be downloaded at: https://www.mdpi.com/article/10.3390/polym14132658/s1, Scheme S1: Illustration of the phosphorylation reactions of the bromoacetic acid with the triphenyl phosphine, Figure S1: Liquid ^1^H, ^13^C and ^31^P NMR of v-PBC, Figure S2: Liquid 1H, ^13^C and ^31^P NMR of v-PBH, Figure S3: Liquid ^1^H and ^31^P NMR of Me-PBC, Figure S4: Liquid 1H, ^13^C and ^31^P NMR of PBH, Figure S5: The FT-IR spectrum of POP-PBC and v-PBC, Figure S6: The FT-IR spectrum of POP-PBH and v-PBH, Figure S7: N_2_ sorption isotherm of POP-PPh_3_-COOH, Figure S8: The TG isotherms of POP-PBC and POP-PBH, Figure S9: CO_2_ sorption isotherm and the Qst of POP-PPh_3_-COOH, Figure S10: The FT-IR spectrum of POP-PBC and the catalyst after recycle for 5 times POP-PBC-5th, Figure S11: The catalytic activities of the heterogeneous catalyst POP-PBC and POP-PBH in the cycloaddition of CO_2_ with different epoxide substrates. Reaction conditions: epoxide (10 mmol), 60 °C for 48 h, Figure S12: The proposed mechanism of the cycloaddition of epoxide and CO_2_ into cyclic carbonate catalyzed by the heterogeneous catalyst POP-PBC, Figure S13: The crude product NMR of cycloaddition reaction over POP-PBC, Table S1: The textural parameters of various polar groups functionalized hierarchical porous organic polymers, Table S2: CO_2_ adsorption performances over various porous materials, Table S3: Comparison of Various Catalysts Proposed for Cycloaddition of CO_2_ with Epichlorohydrin. References [60,61,62,63,64,65,66] are cited in the supplementary materials.
