# Supplementary material for: Functional Porous Ionic Polymers as Efficient Heterogeneous Catalysts for the Chemical Fixation of CO2 under Mild Conditions
Source: Polymers (Basel). 2022 Jun 29;14(13):2658. doi: 10.3390/polym14132658 (PMC9269538; doi:10.3390/polym14132658)
Supplement: Supplementary file 1 [file polymers-14-02658-s001.zip › polymers-1729695-supplementary.pdf]

Supplementary Materials

Functional Porous Ionic Polymers as Efficient Heterogeneous Catalysts for the Chemical Fixation of CO_2 under Mild Conditions

Zhifeng Dai [1,2,3,*], Yang Long [2], Jianliang Liu [2], Yuanfei Bao [2], Liping Zheng [2], Jiacong Ma [2], Jiayi Liu [2], Fei Zhang [4,*], Yubing Xiong [2,3,*] and Ji-Qing Lu [1,*]

1 Key Laboratory of the Ministry of Education for Advanced Catalysis Materials, College of Chemistry and Life Science, Zhejiang Normal University, Jinhua 321004, China
2 Key Laboratory of Surface & Interface Science of Polymer Materials of Zhejiang Province, Department of Chemistry, College of Science, Zhejiang Sci-Tech University, Hangzhou 310018, China; 202130107313@mails.zstu.edu.cn (Y.L.); 202030107252@mails.zstu.edu.cn (J.L.); 202030107214@mails.zstu.edu.cn (Y.B.); 202120104190@mails.zstu.edu.cn (L.Z.); 2020326602050@mails.zstu.edu.cn (J.M.); 2020326602068@mails.zstu.edu.cn (J.L.)
3 Longgang Institute of Zhejiang Sci-Tech University, Wenzhou 325802, China
4 Institute of Advanced Materials, College of Chemistry and Chemical Engineering, Jiangxi Normal University, Nanchang 330022, China
* Correspondence: daizhif@zstu.edu.cn (Z.D.); fzhang@jxnu.edu.cn (F.Z.); ybxiong@zstu.edu.cn (Y.X.); jiqinglu@zjnu.cn (J.-Q.L.)

Experimental

Synthesis of tri(4-vinylphenyl) phosphine. PCl_3 (3.4 g, 25 mmol) in 20 mL THF was added into the solution of (4-vinylphenyl)magnesium bromide (18.3 g, 100 mmol, synthesized from the 4-bromostyrene and magnesium) at 0 °C under N_2, stirred at room temperature overnight, and then quenched by the addition of 50 mL of saturated NH_4Cl solution. After extraction with ethyl acetate, washing with brine, drying over $MgSO_4$, filtering, concentrating under vacuum, and purifying by flash column chromatography on silica gel (volume ratio of petroleum ether to EtOAc at 40), the tri(4-vinylphenyl) phosphine as white solid (5.8 g, 68.0% yield) was finally obtained.

Synthesis of Carboxymethyl-tris(4-vinylphenyl)phosphonium bromide (v-PBC). In a typical round, a solution of tri(4-vinylphenyl) phosphine (5 mmol) and bromoacetic acid (5 mmol) in acetone (20 mL) was stirred at 343 K for 48 h. After then, the mixture was cooled to room temperature and the solvent was removed under vacuum. The residue was dissolved in little amount of CH_2Cl_2 and the product was separated by addition of the mixture to an excessive amount of ethyl acetate. The solid was filtrated, washed with ethyl acetate for several times and dried under vacuum. The product was obtained as a pale yellow solid in 75.3% yield.

Synthesis of Carboxymethyl-tris(4-vinylphenyl)phosphonium bromide (v-PBH). In a typical round, a solution of tri(4-vinylphenyl) phosphine (5 mmol) and 2-bromoethanol (5 mmol) in acetone (20 mL) was stirred at 70 °C for 48 h. After then, the mixture was cooled to room temperature the solvent was removed under vacuum. The residue was dissolved in little amount of CH_2Cl_2 and the product was precipted by addition of the mixture to an excessive amount of diethyl ether. The solid was filtrated, washed with diethyl ether for several times and dried under vacuum. The product was obtained as a light yellow solid in 85.6% yield.

Synthesis of (carboxymethyl)tri-*p*-tolylphosphonium bromide (Me-PBC). bromoacetic acid (276 mg, 1.8 mmol) and tris(4-methylphenyl) phosphine (0.5 g, 1.64 mmol) were dissolved in 10 mL of toluene and stirred at 60 °C under N_2 atmosphere for 48 h. After the reaction, the mixture was cooled to room temperature and the solid was filtrated, washed with diethyl ether, and dried under vacuum. The product was obtained as white solid (576 mg, 76.8% yield).

Synthesis of (2-hydroxyethyl)triphenylphosphonium bromide (PBH). 2-bromoethanol (226 mg, 1.80 mmol) and trisphenyl phosphine (431 g, 1.64 mmol) was dissolved in 10 mL of acetone, stirring at

60 °C under N_2 atmosphere for 48 h. After the reaction, the mixture was cooled to room temperature and the solvent was removed under vacuum. Then the residue was dissolved in a little CH_2Cl_2 and the product was separated by the addition of excessive amount of diethyl ether. The solid was filtrated, washed with diethyl ether, and dried under vacuum. The product was obtained as white solid (523 mg, 82.3% yield).

Synthesis of carboxylic acid functionalized porous organic polymer (POP-PPh$_3$-COOH). For the synthesis of POP-PPh$_3$-COOH, 300 mg of POP-PPh$_3$ (0.88 mmol) and 244.5 mg of bromoacetic acid (1.76 mmol) were dispersed in 10 mL of toluene with stirring, then the mixture was heated to 80 °C for 48 h under N_2. After cooling to room temperature, the solid was filtered and washed with ethyl acetate for several times. After dried under vacuum for 12 h, the product POP-PPh$_3$-COOH was obtained as orange solid (370.1 mg, 87.6 % yield). The content of Br^- species in the porous organic polymer was calculated to be 11.2 wt% accordingly.

The amount of Br^- species in the POP-PBC and POP-PBH were calculated from the following equation:

$$w(Br^-) = \frac{M_{Br}}{M_p}$$

In which the M_P represents the molecular weight of the phosphonium monomers. M_{Br} represents the atomic weight of the Br^- species. The amount of Br^- species in the POP-PPh$_3$-COOH was calculated from the following equation:

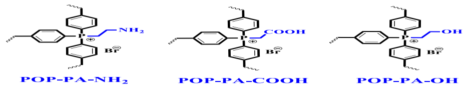

In which the mp and ms represent the amount of the yield products and the substrates used for the phosphonium reaction, respectively. M_{PG} represents the molecular weight of the decorated polar group (PG). M_{Br} represents the atomic weight of the Br^- species.

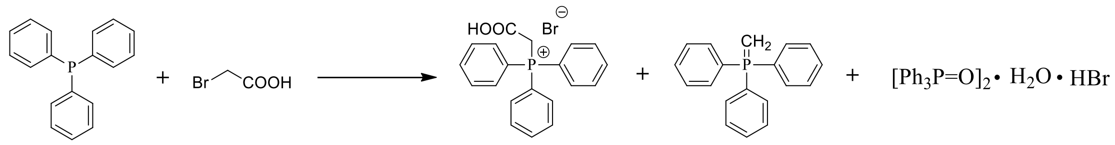

Scheme S1. Illustration of the phosphorylation reactions of the bromoacetic acid with the triphenyl phosphine.

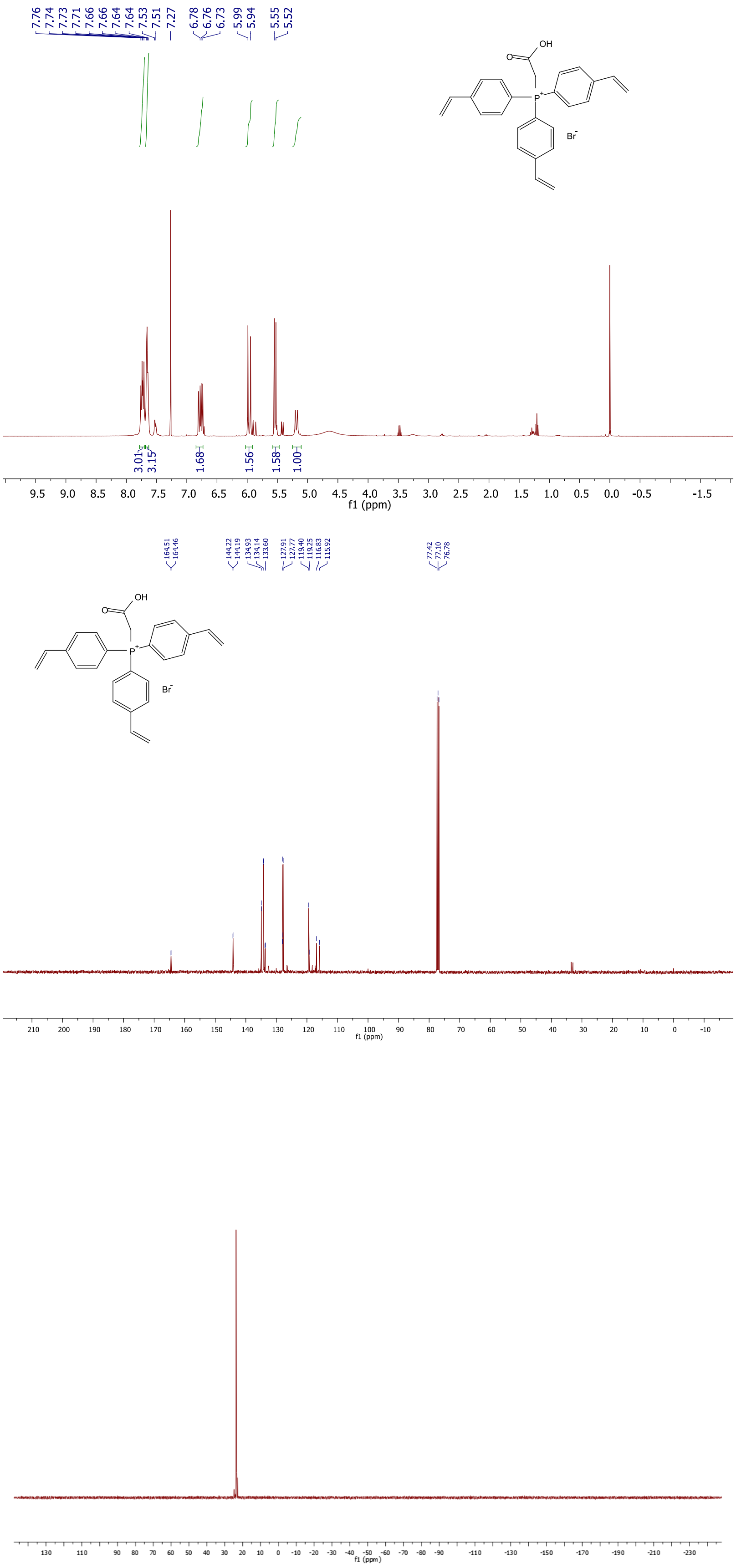

Figure S1. Liquid ^{1}H, ^{13}C and ^{31}P NMR of v-PBC.

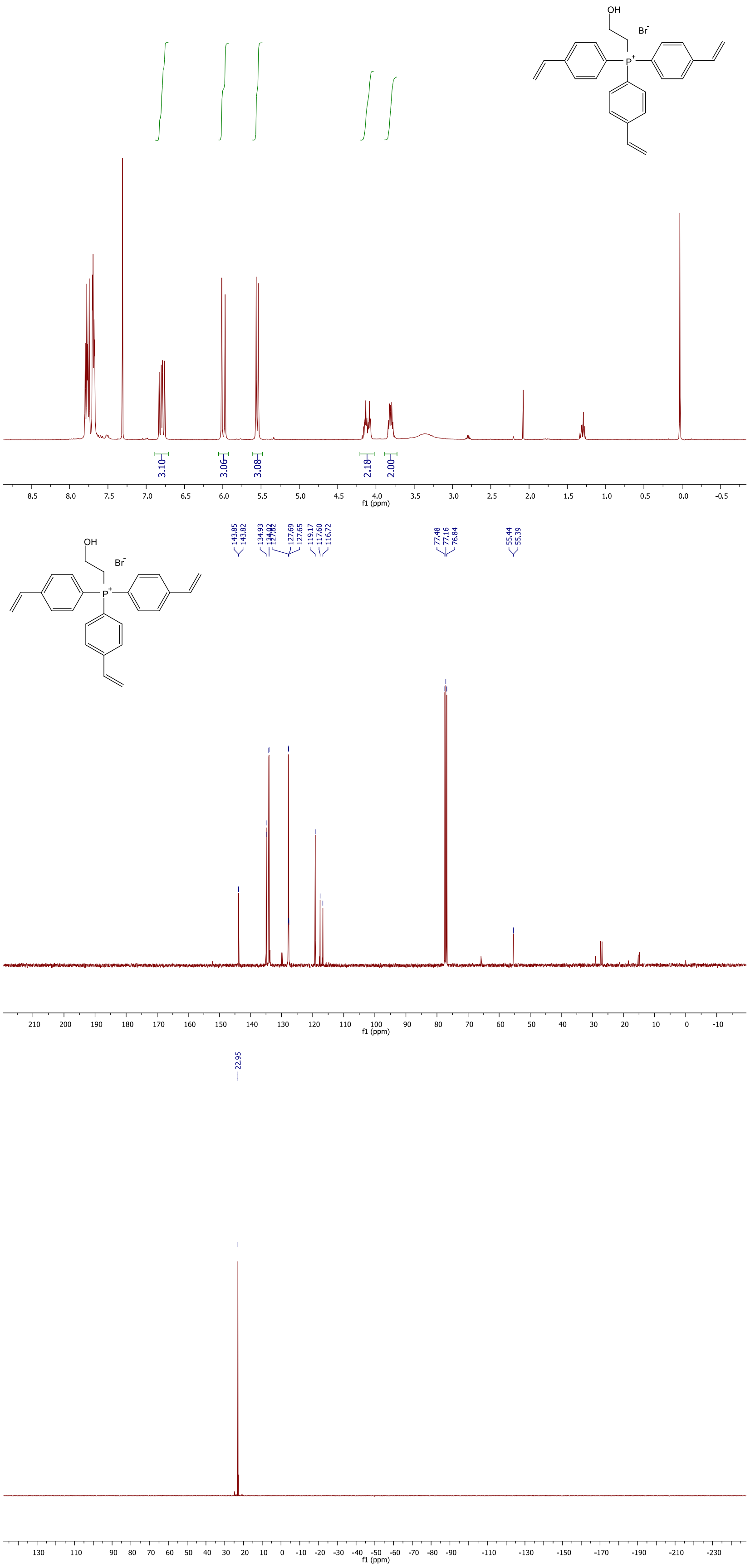

Figure S2. Liquid ^{1}H, ^{13}C and ^{31}P NMR of v-PBH.

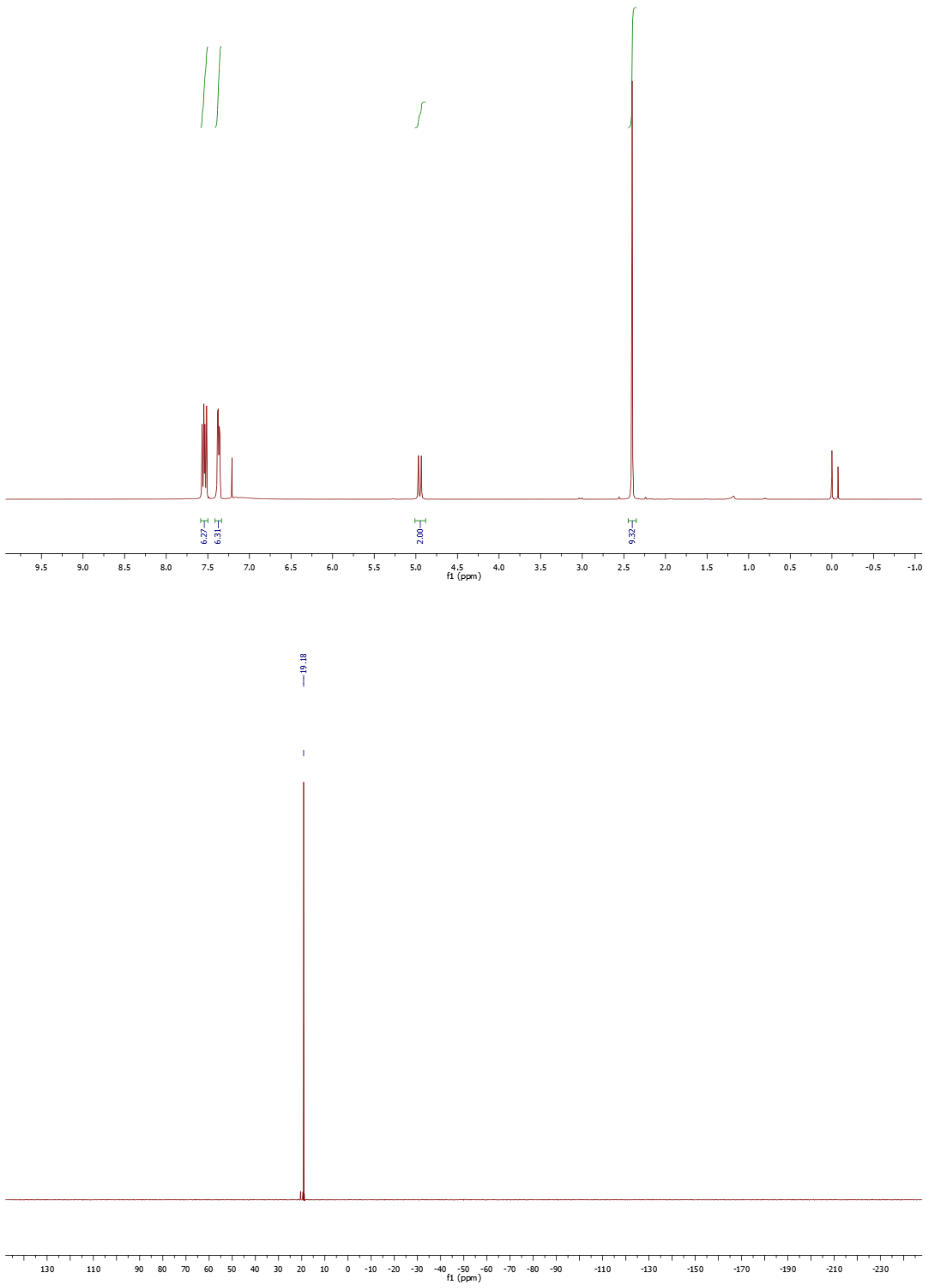

Figure S3. Liquid ^{1}H and ^{31}P NMR of Me-PBC.

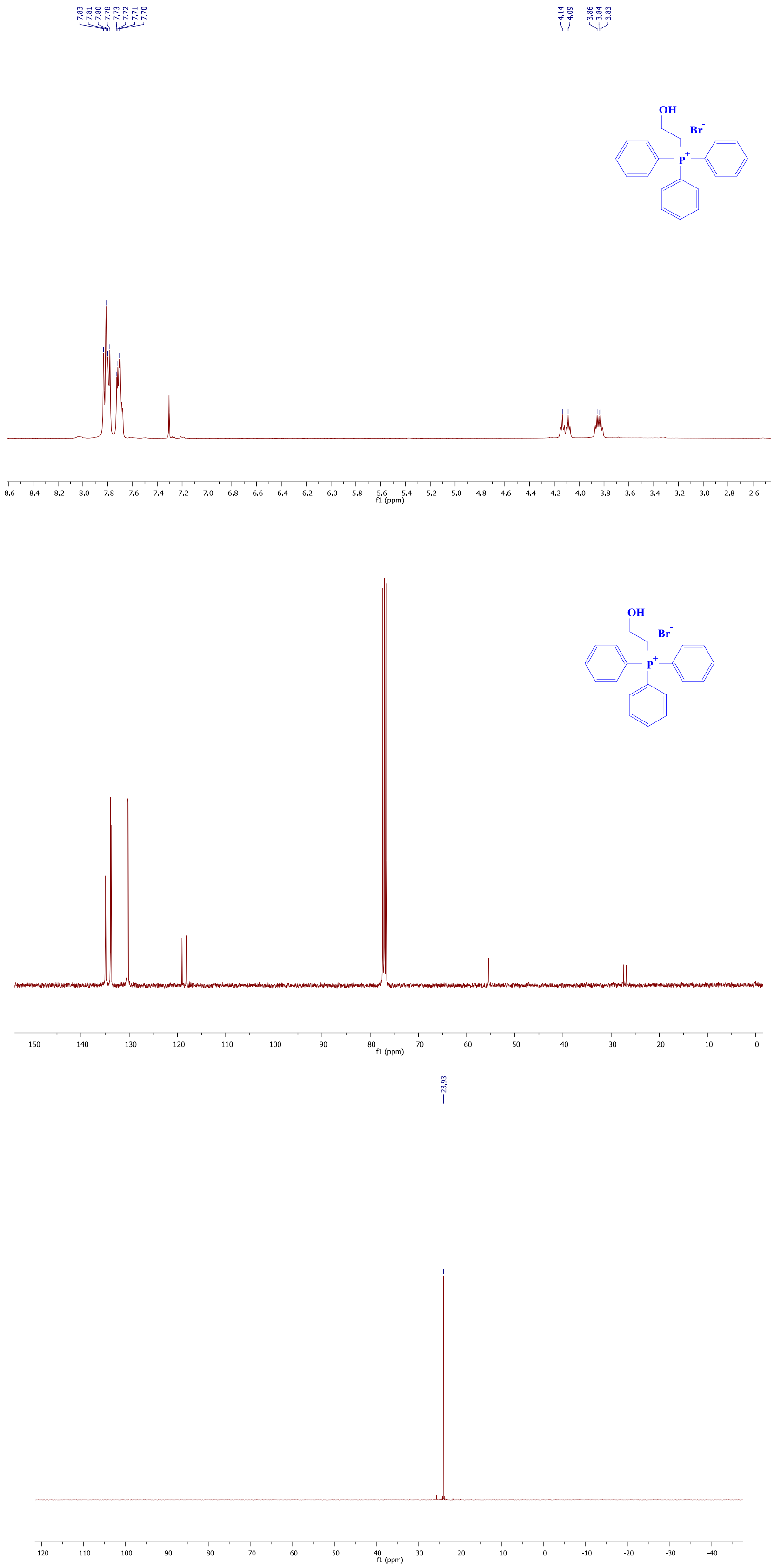

Figure S4. Liquid ^{1}H, ^{13}C and ^{31}P NMR of PBH.

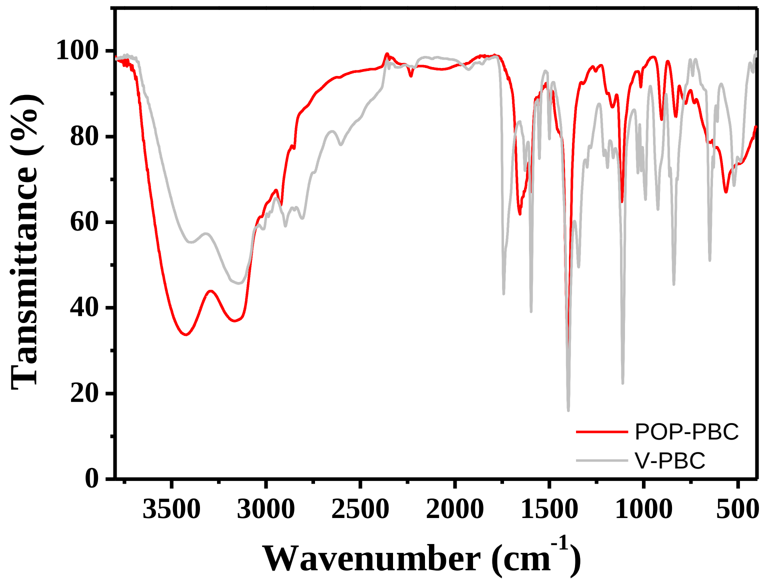

Figure S5. The FT-IR spectrum of POP-PBC and v-PBC.

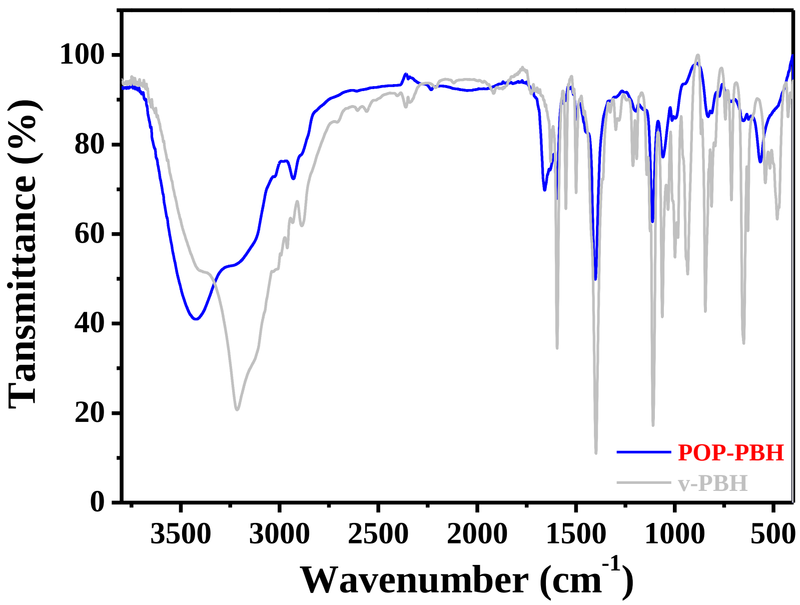

Figure S6. The FT-IR spectrum of POP-PBH and v-PBH.

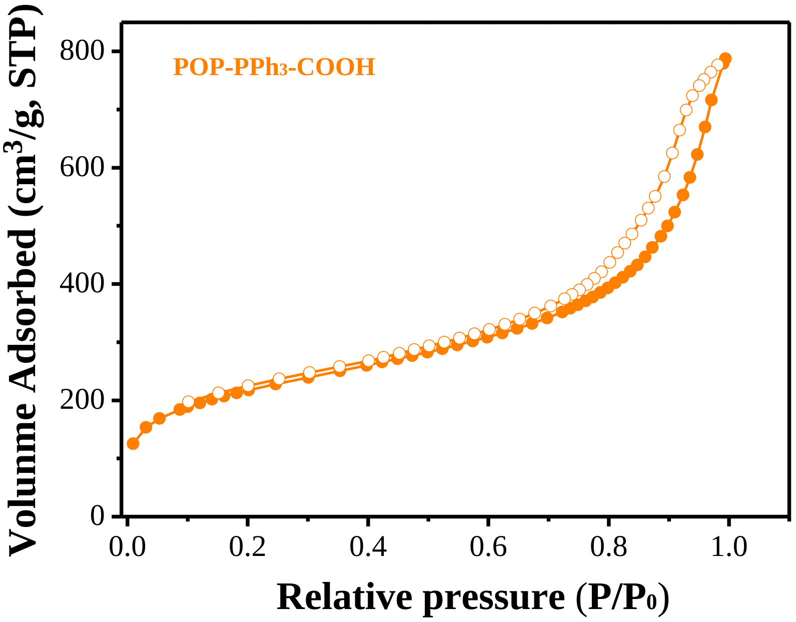

Figure S7. N_2 sorption isotherm of POP-PPh_3-COOH.

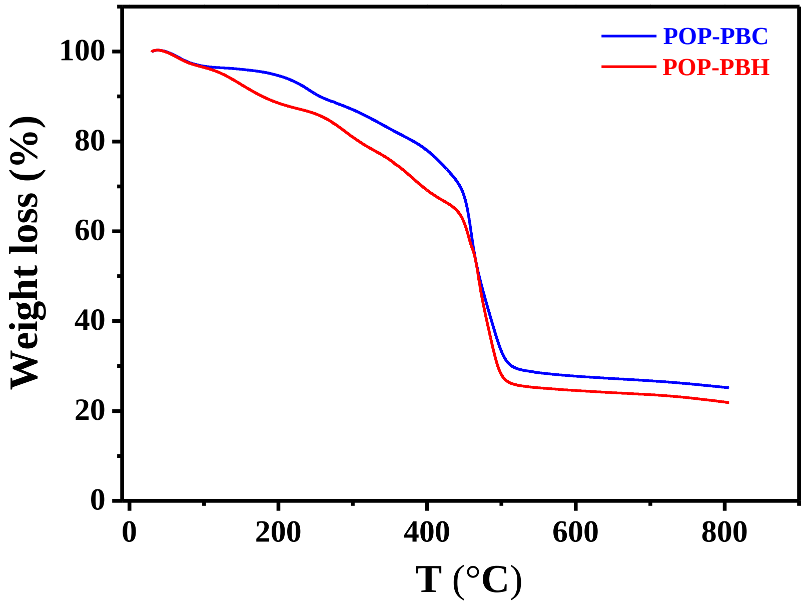

Figure S8. The TG isotherms of POP-PBC and POP-PBH.

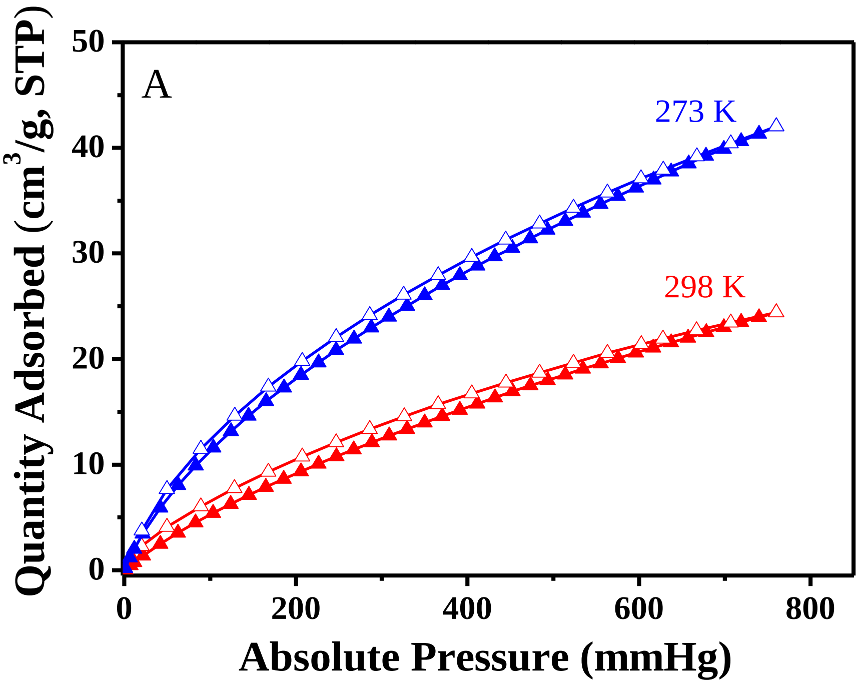

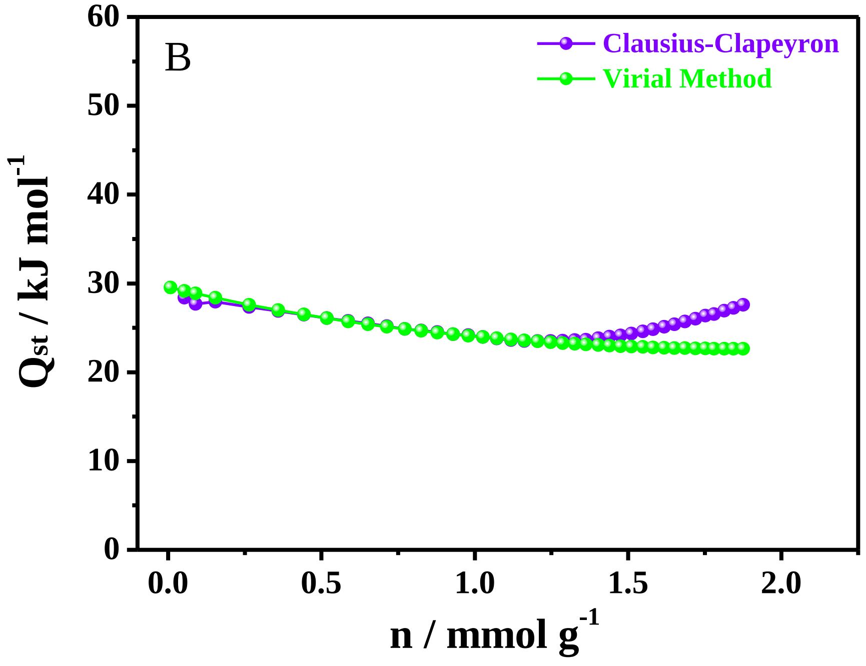

Figure S9. CO_2 sorption isotherm and the Qst of POP-PPh_3-COOH.

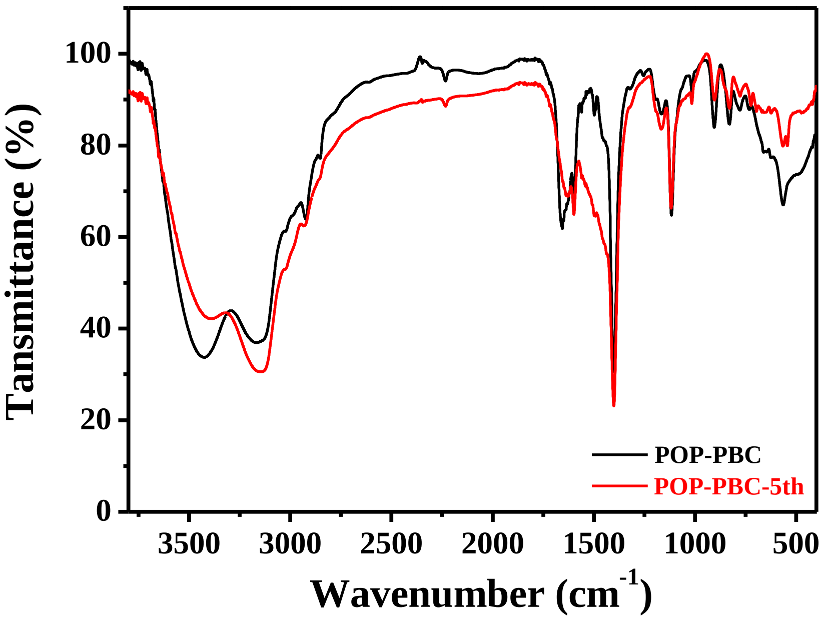

Figure S10. The FT-IR spectrum of POP-PBC and the catalyst after recycle for 5 times POP-PBC-5th.

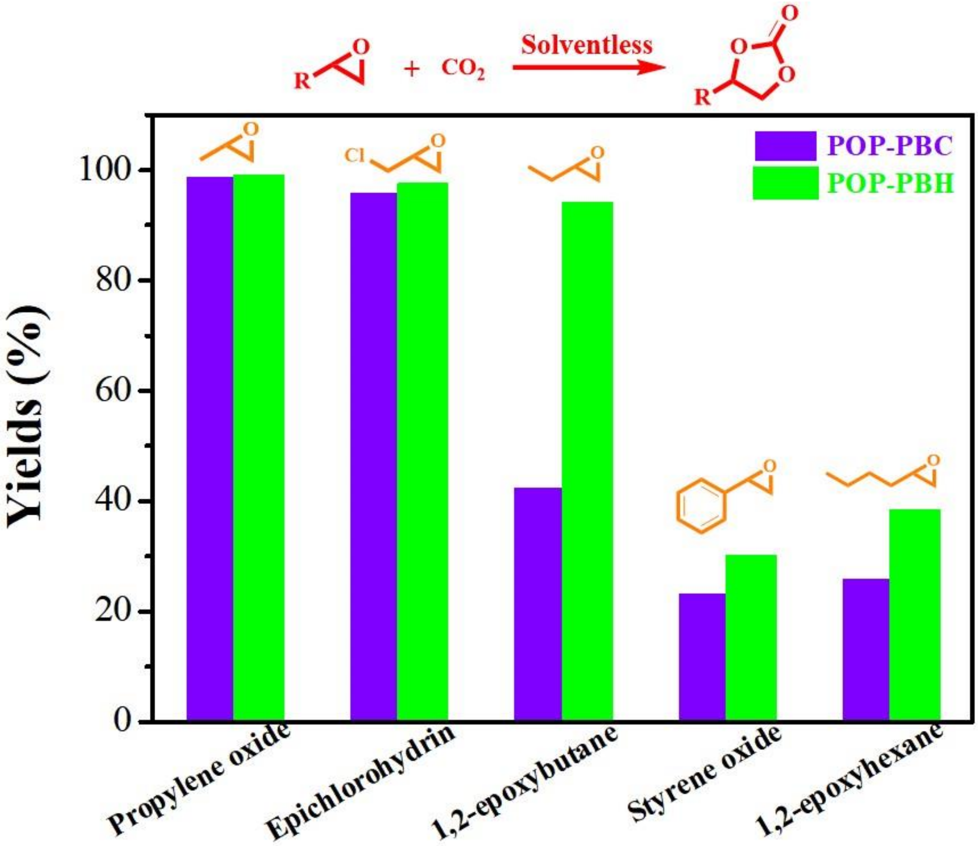

Figure S11. The catalytic activities of the heterogeneous catalyst POP-PBC and POP-PBH in the cycloaddition of CO_2 with different epoxide substrates. Reaction conditions: epoxide (10 mmol), 60 °C for 48 h.

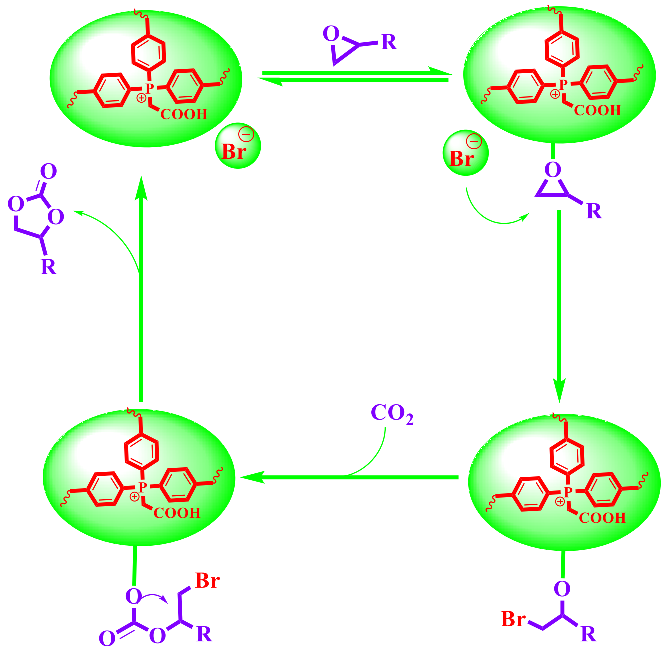

Figure S12. The proposed mechanism of the cycloaddition of epoxide and CO_2 into cyclic carbonate catalyzed by the heterogeneous catalyst POP-PBC.

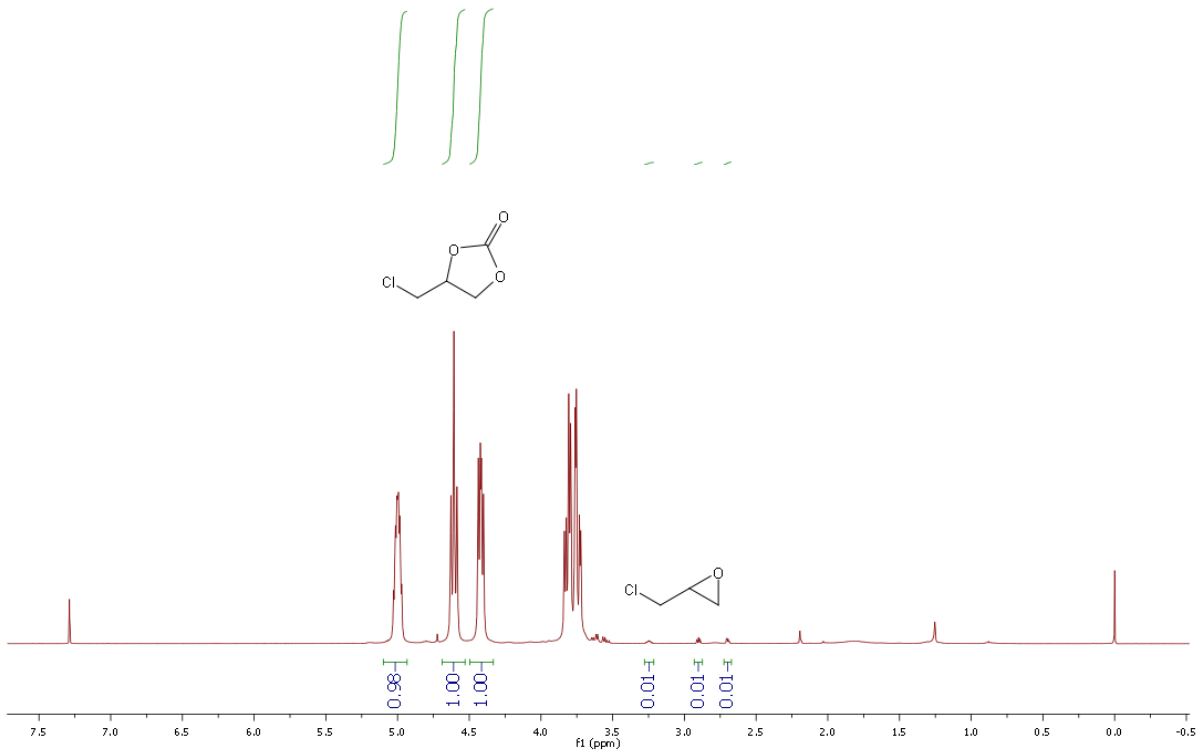

Figure S13. The crude product NMR of cycloaddition reaction over POP-PBC.

Table S1. The textural parameters of various polar groups functionalized hierarchical porous organic polymers.

Polymers	BET (m^2/g)	Pore Volume (cm^3/g)	Element content
			Br (%)
POP-PBC	772	0.57	16.5
POP-PBH	643	1.3	16.9
POP-PPh_3-COOH	777	1.2	10.9

Table S2. CO_2 adsorption performances over various porous materials.

Material	SA_{BET} (m²/g)	Adsorption capacity (mg/g)		Qst (kJ/mol)	Ref.
		273 K	298 K		
POP-PBC	772	127	79	31.7	This work
POP-PBH	692	116	70	30.6	This work
P-POF-Zn	639	85	67	25	[24]
CAP	845	134	86	35.4	[24]
CAP-DAP	517	153	99	46.6	[54]
DB-Tp	916	109	65	28	[55]
POP-PBnCl-TPPMg-4	411	55	37	28.8	[29]
POP-PBnCl-TPPMg-12	462	82	50.6	31.8	[29]
POP-BPy	1123	130	70	28.0	[41]
POP-TPP	1200	--	58.3	--	[26]
BILP-1	1172	188	131	26.5	[60]
PAF-1	5640	91	--	15.6	[61]
CMP-0	1018	92.4	53.2	--	[62]
TNCMP-2	995	115	64	--	[62]
SMPI-10	112	139	82	--	[63]
TPI-1	809	108	55	34.4	[64]
FJC-1	1726	126	81	20.7	[65]
Co-CMP	965	--	79.3	--	[66]

Table S3. Comparison of Various Catalysts Proposed for Cycloaddition of CO_2 with Epichlorohydrin.

Catalysts	Epichlorohydrin	Time	T (°C)	CO_2 pressure	Conv. (%)	Sel. (%)	Ref.
POP-PBC (48.0 mg)	10 mmol	48 h	60	0.1 MPa	96.2	99.0	This work
POP-PBH (46.6 mg)	10 mmol	48 h	60	0.1 MPa	97.2	99.0	This work
PAD-3 (100 mg)	5 mmol	24 h	70	0.1 MPa	98.0	99.0	[56]
PGDBr-5-2OH (50 mg)	5 mmol	24 h	70	0.1 MPa	91	99.0	[45]
PIL-1 (10 mg)	5 mmol	15 h	100	1 MPa	61.2	99.0	[57]
MIL−101(Cr)−TSIL (100 mg)	15 mmol	2 h	110	2 MPa	99.0	81	[58]
IL@MIL-101−SO_3H (80 mg)	10.8 mmol	24 h	90	1 MPa	74	99.0	[59]
